# An improved YOLOv5s model for assessing apple graspability in automated harvesting scene

**DOI:** 10.3389/fpls.2023.1323453

**Published:** 2023-12-11

**Authors:** Huibin Li, Peng Yang, Huaiyang Liu, Xiang Liu, Jianping Qian, Qiangyi Yu, Changxing Geng, Yun Shi

**Affiliations:** ^1^ State Key Laboratory of Efficient Utilization of Arid and Semi-arid Arable Land in Northern China, Institute of Agricultural Resources and Regional Planning, Chinese Academy of Agricultural Sciences, Beijing, China; ^2^ Agricultural Algorithm Research Department, Suzhou Zhongnong Digital Intelligence Technology Co., Ltd, Suzhou, China; ^3^ School of Mechanical and Electrical Engineering, Soochow University, Suzhou, China

**Keywords:** apple harvesting, BYOL, attention mechanism, occlusion detection, YOLOv5S

## Abstract

**Introduction:**

With continuously increasing labor costs, an urgent need for automated apple- Qpicking equipment has emerged in the agricultural sector. Prior to apple harvesting, it is imperative that the equipment not only accurately locates the apples, but also discerns the graspability of the fruit. While numerous studies on apple detection have been conducted, the challenges related to determining apple graspability remain unresolved.

**Methods:**

This study introduces a method for detecting multi-occluded apples based on an enhanced YOLOv5s model, with the aim of identifying the type of apple occlusion in complex orchard environments and determining apple graspability. Using bootstrap your own atent(BYOL) and knowledge transfer(KT) strategies, we effectively enhance the classification accuracy for multi-occluded apples while reducing data production costs. A selective kernel (SK) module is also incorporated, enabling the network model to more precisely identify various apple occlusion types. To evaluate the performance of our network model, we define three key metrics: AP_GA_, AP_TUGA_, and AP_UGA_, representing the average detection accuracy for graspable, temporarily ungraspable, and ungraspable apples, respectively.

**Results:**

Experimental results indicate that the improved YOLOv5s model performs exceptionally well, achieving detection accuracies of 94.78%, 93.86%, and 94.98% for AP_GA_, AP_TUGA_, and AP_UGA_, respectively.

**Discussion:**

Compared to current lightweight network models such as YOLOX-s and YOLOv7s, our proposed method demonstrates significant advantages across multiple evaluation metrics. In future research, we intend to integrate fruit posture and occlusion detection to f]urther enhance the visual perception capabilities of apple-picking equipment.

## 1 Introduction

During the apple maturation season, orchard managers typically employ a significant temporary workforce to ensure the timely harvesting and sale of the apples. However, in recent years, escalating labor costs and the scarcity of manpower have posed significant challenges for these managers (Liu et al., 2019). Consequently, there is an increasing demand for automated apple-picking equipment in the agricultural sector, which represents a pivotal opportunity for the development of such technology. Over the past few decades, apple-picking equipment has garnered substantial attention from both domestic and international researchers (Li et al., 2022). While vision-based apple-picking control technologies have experienced rapid advancement, the hand–eye coordination efficiency of the equipment remains sub-optimal and has been identified as a key factor affecting its performance (Jiao et al., 2020). Occlusion is considered one of the primary challenges in improving visual control technology for apple-picking. This factor has a negative impact, as occlusion by leaves, branches, or other apples can prolong the apple identification time and reduce accuracy.

At present, apple-picking equipment can harvest apples that are unobstructed or merely occluded by leaves; however, apples concealed by branches or other apples pose a significant challenge. During automated harvesting,if the equipment cannot discern the graspability of an apple based on its occlusion status, the equipment may fail to grasp the apple or even become damaged, severely compromising its safety. In this study, the graspability of apples refers to whether the apple’s growing environment is suitable for robotic hands to safely harvest them (Yan et al., 2021).To enhance the selective grasping capabilities of apple-picking equipment, it is imperative for network models to discern occlusions produced by branches, leaves, and apples. Recent deep learning-based apple identification network model research has predominantly focused on the DasNet (Kang and Chen, 2019; Kang and Chen, 2020), YOLO (Dean et al., 2019; Wu et al., 2021; Wang et al., 2022), R-CNN (Dandan and Dongjian, 2019; Zhang et al., 2020), and Mask R-CNN (Jia et al., 2020; Chu et al., 2021) series of models. However, most studies have conducted apple identification through single-class recognition, overlooking the impacts of occlusions on harvesting. To mitigate risks during harvesting and boost operational efficiency, apple-picking equipment should be capable of precisely detecting various apple occlusion scenarios prior to harvesting, subsequently determining the graspability of apples; however, such detection methods are inherently more challenging, as they rely on subtle features based on the apple’s local position (Minervini et al., 2016), making these fine-grained features elusive.

In conducting multi-occlusion apple detection, it is imperative to first compile a comprehensive data set representing various apple occlusion types, ensuring that these data accurately depict a myriad of occlusion scenarios. However, the compilation of such a data set is not only time-consuming and costly, but also susceptible to mislabeling of occlusion categories, which can compromise the accuracy of the final model. Furthermore, many network models, burdened by their substantial weights, exhibit sub-par real-time performance. In contrast, lightweight models, while boasting rapid computational speeds, often suffer from diminished recognition accuracy. To address these challenges, there is a pressing need to explore techniques centered on model-based label generation, parameter optimization, and architectural design. Wang et al. proposed an R-FCN network model based on ResNet-34 that adeptly identifies apples in the presence of overlap, leaf occlusion, and surface shadows, achieving recognition recall and accuracy rates of 85.7% and 95.1%, respectively (Dandan and Dongjian, 2019). Jia (Jia et al., 2020) introduced a lightweight modification into Mask R-CNN by integrating ResNet and DenseNet, and the model’s detection precision and recall rates reached 97.31% and 95.70%, respectively. However, the model’s detection speed still fell short of real-time detection requirements. Addressing this, Kuznetsova (Kuznetsova et al., 2020) proposed a pre-processing and post-processing approach based on YOLOv3, achieving a rapid detection speed of 19 ms per frame. Yan (Yan et al., 2022) designed the Bottleneck CSP-B module and an SE attention module based on YOLOv5m, making preliminary strides in detecting apple occlusion scenarios; nevertheless, instances of misidentification or outright non-recognition of apples were observed. Kang (Kang and Chen, 2020) introduced LedNet, which offers extensive data labeling capabilities, with the aim of enhancing fruit detection precision. While existing studies have made progress in terms of apple detection, there remains a pivotal need to address misidentification issues in multi-occlusion apple scenarios. This factor is crucial to ensure the precise determination of apple graspability and fulfill the demands of apple harvesting operations.

For this study, mature apples that remained unharvested in an orchard were selected as the subjects of investigation, and an occlusion-aware apple detection method based on an enhanced YOLOv5s model was proposed. Utilizing the results from this multi-occlusion apple detection method, the graspability of the apples was further assessed. To effectively reduce the need for annotations, minimize data preparation costs, and improve the performance of the YOLOv5s backbone, a training scheme based on self-supervised learning and knowledge transfer was employed. Additionally, the selective kernel module was integrated, enabling the refined YOLOv5s to more accurately identify apples with multiple occlusions, thereby enhancing the apple harvesting equipment’s ability to determine apple graspability. This research offers a viable solution for precisely discerning apple graspability and has significant implications for improving the efficiency and safety of apple harvesting equipment.

## 2 Materials and methods

### 2.1 Apple orchard environment

**Figure 1:**
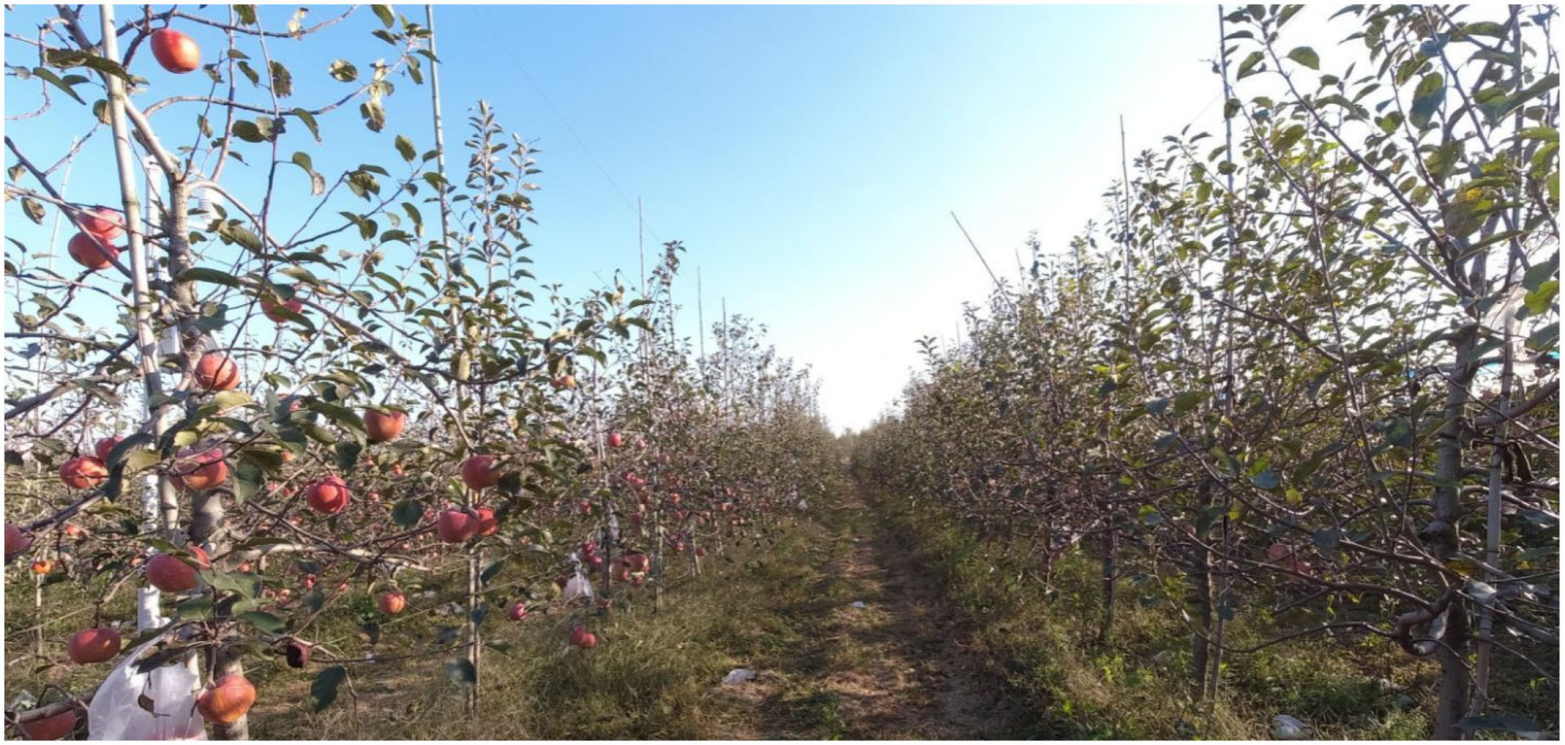
Planting scene of Zoumaling Orchard in Biguo Town, Zhaoyuan County.

Yantai City, located in the northeastern part of the Shandong Province, has geographical coordinates of 119°34′E to 121°57′E longitude and 36°16′N to 38°23′N latitude. Recognized as the birthplace of modern apple cultivation in China, Yantai is also among the country’s primary apple-growing cities. The apple image data utilized in this study were collected in October 2021 from the Zoumaling Orchard in Biguo Town, Zhaoyuan County, Yantai City. This orchard utilizes a modern spindle-shaped planting structure. The apple trees are spaced approximately 3.5 m apart, with a distance of about 1.5 m between individual trees and an average height of around 3.5 m, as depicted in **Figure 1**. During the apple maturation phase, the apples display a vibrant red hue, are densely clustered, and become relatively large, with an average weight of 319 g per apple. Prior to harvesting, the apple trees were sprayed with defoliants by orchard management personnel, which expedite the shedding of leaves. Consequently, by the time of apple maturation, fewer leaves remained on the apple trees, revealing a more pronounced presence of branches and resulting in a sparse canopy pattern. This distribution of branches and leaves not only provides the apples with increased sunlight exposure, but also presents a realistic scenario for research into the automated harvesting of apples.

### 2.2 Data collection and annotation

#### 2.2.1 Data collection

**Figure 2:**
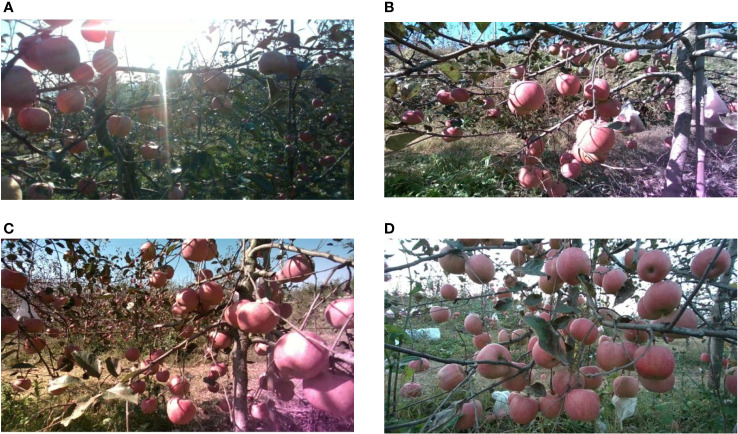
Images under different lighting conditions. **(A)** Back-lit image. **(B)** front-lit image. **(C)** side-lit image. **(D)** low-light image.

An Intel D455 camera was employed to capture images at a range of 0.3 to 1.0 m from the apple trees. To ensure diversity in the captured images, the potential effects of varying weather and lighting conditions on the images were thoroughly considered. Images were taken during three distinct periods—morning, noon, and afternoon—and under both clear and cloudy weather conditions. These images were captured under various lighting modes, including front-lit, back-lit, and side-lit, as illustrated in **Figure 2**. In total, 5000 images with a resolution of 1280 × 720 pixels were collected, all of which were saved in the PNG format. After eliminating images with high redundancy, a final set of 2800 high-quality apple images were retained.

#### 2.2.2 Data annotation

**Figure 3:**
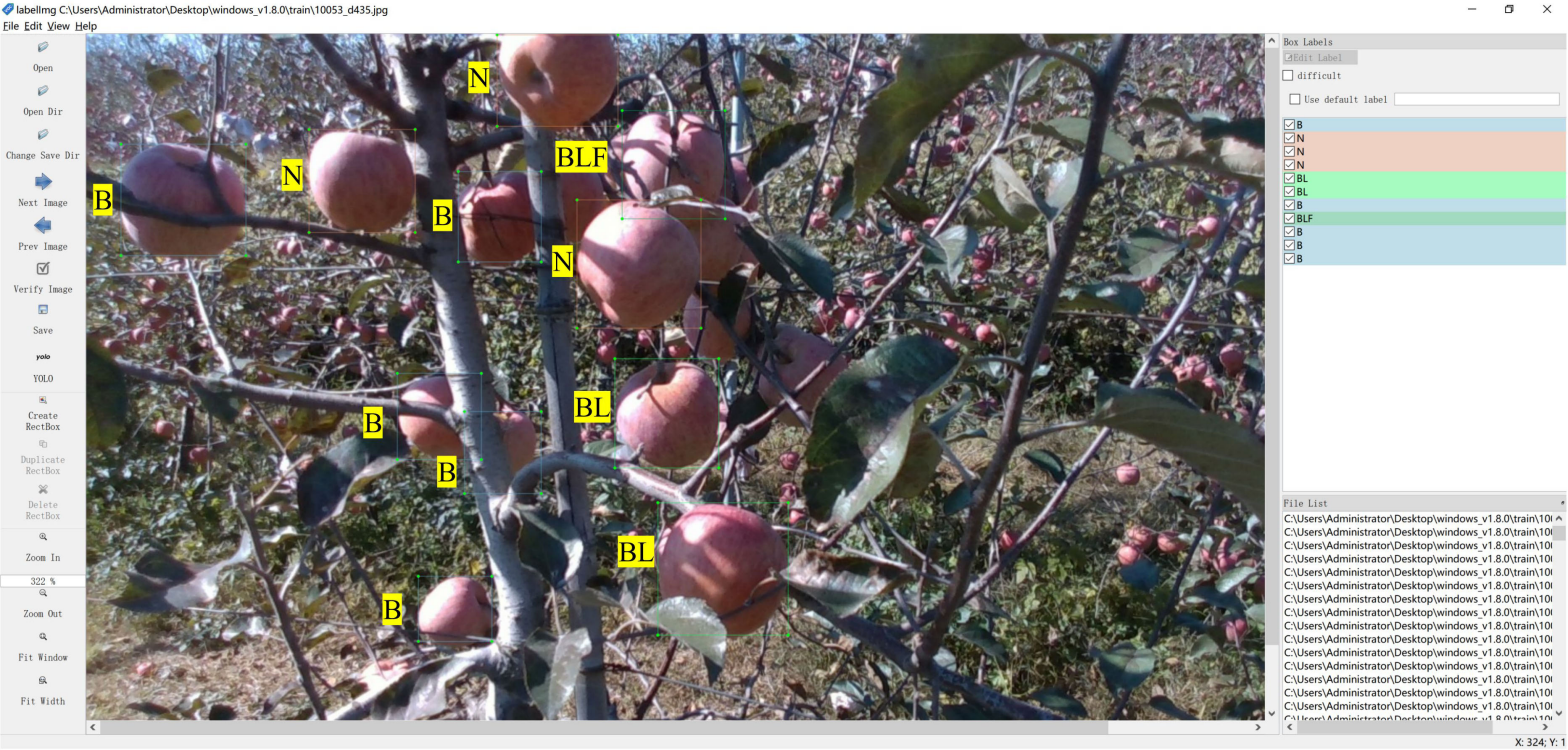
Annotation results for various occluded apples in an image using the LabelImg. N stands for No occlusion, B stands for Branch occlusion, BL stands for Leaf and Branch occlusion, BLF stands for Leaf, Branch, and Apple occlusion.

Meticulous annotation of the images was conducted based on occlusion of the apple surfaces by branches, leaves, and other apples. All occlusion scenarios within the images were categorized into eight classes: No occlusion (N), Leaf occlusion (L), Apple occlusion (F), Branch occlusion (B), Leaf and Apple occlusion (LF), Leaf and Branch occlusion (BL), Branch and Apple occlusion (BF), and combined Leaf, Branch, and Apple occlusion (BLF). The LabelImg annotation software was employed (Zhuk et al., 2015), with labels generated in txt format. The results of the various occlusion annotations are depicted in **Figure 3**. From the perspective of actual apple harvesting operations, apples were classified into three categories based on their occlusion status: apples categorized as N or L were deemed to be Graspable Apples (GA), as the harvesting process remains unaffected when apples are either unobstructed or solely obstructed by leaves; apples categorized as F or LF were categorized as Temporarily Ungraspable Apples (TUGA) as, once the apples obstructing the surface are harvested, these apples can become subsequent grasping targets; and apples categorized as B, BL, BF, or BLF were classified as Ungraspable Apples (UGA),primarily due to branch obstructions, which could potentially damage the apples or the harvesting equipment if direct harvesting were attempted.

As detailed in **Table 1**, the data set contained a total of 36,803 annotated bounding boxes, among which ungraspable apples constituted the majority, accounting for 51.5% of total annotations. Graspable apples represented 42.0% of the total, while temporarily ungraspable apples made up 7.5%. The annotated results were divided into training, validation, and test sets at a ratio of 7:1:2, serving the purposes of network model training, optimization, and performance evaluation, respectively. During the training process, data augmentation techniques were employed, primarily involving the addition of noise to and forming mosaics of the images, as well as adjustments to contrast and brightness.

**Table 1:** Statistics for three types of apple targets.

No. of Apples	No. of Graspable Apples		No. of Temporarily Ungraspable Apples		No. of Ungraspable Apples			
	N	L	F	LF	B	BL	BF	BLF
36803	9856	5597	1936	474	10,579	6405	1296	660

### 2.3 Construction of detection model

#### 2.3.1 Methodology overview

**Figure 4:**
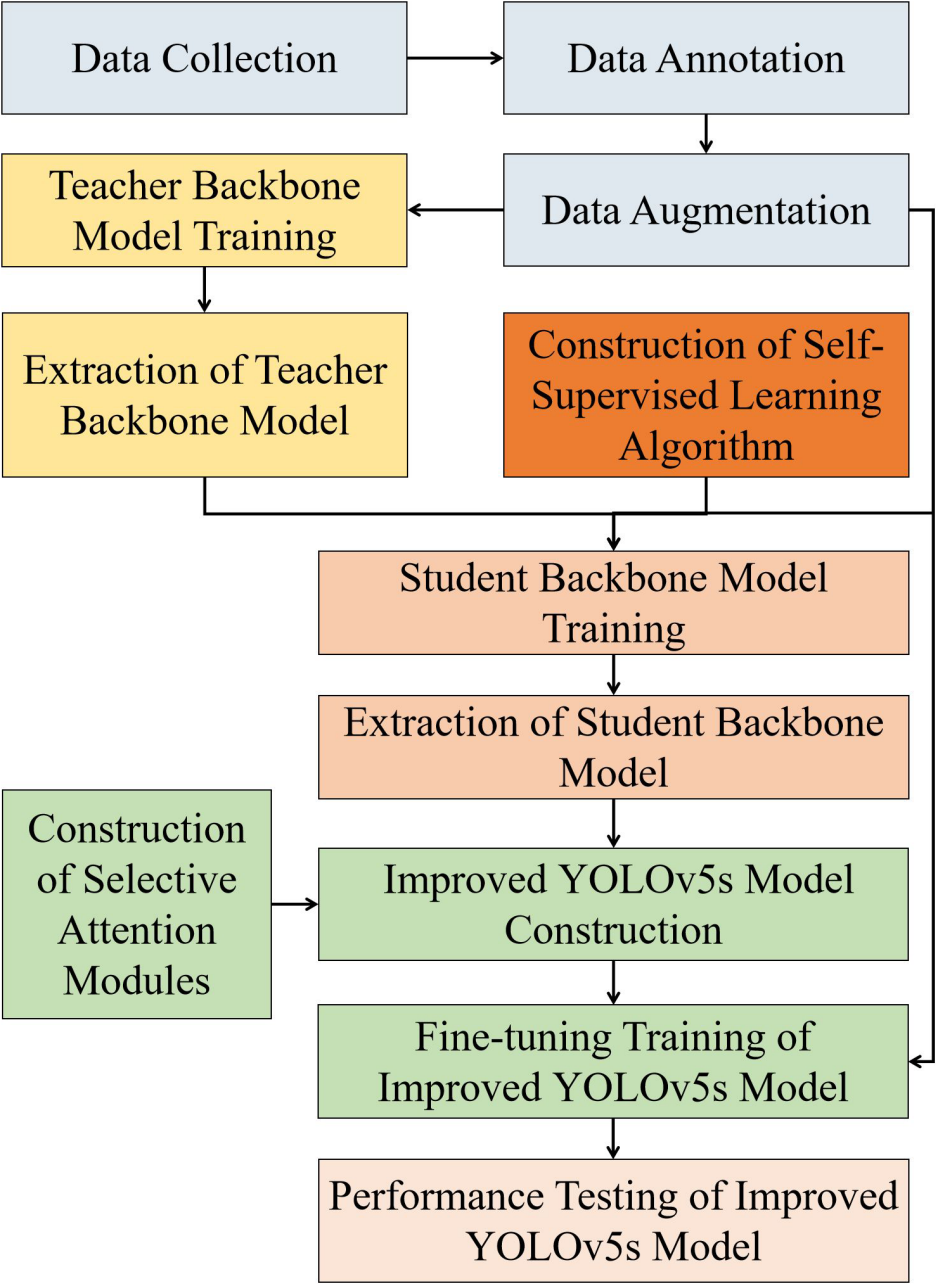
Technological framework of the proposed approach. In the technical framework, the same color represents the same experimental stage, grey boxes represent raw images pre-processing, yellow boxes represent the construction of teacher backbone model, dark yellow boxes represent the construction of self-supervised learning algorithm, light red boxes represent the construction of student backbone model, green boxes represent the improvement process of YOLOv5s model, and light pink represents the performance outputs of the improved YOLOv5s model.

To determine the graspability of the apples, we introduce a detection method for multi-occluded apples based on an enhanced YOLOv5s model. In particular, this method determines the graspability of the fruit based on the occlusion detection results. The technical framework of this method is depicted in **Figure 4**. Initially, data collection, annotation, and augmentation are conducted, establishing an eight-category occluded apple data set. The YOLOv5s model was employed for fully supervised data training, and the backbone of the post-training model was extracted to serve as the teacher backbone model for guided training. Given the data set size constraints, a joint training strategy combining knowledge transfer and self-supervised learning algorithms was devised, primarily aiming to construct a more robust student backbone model. To further optimize YOLOv5s, we integrated the SK module (Li et al., 2019). Ultimately, the student backbone model was utilized to initialize the enhanced YOLOv5s backbone. With the aid of the augmented training set, fully supervised fine-tuning was conducted in order to achieve optimal performance of the improved YOLOv5s model.

#### 2.3.2 Improvement of YOLOv5s

**Figure 5:**
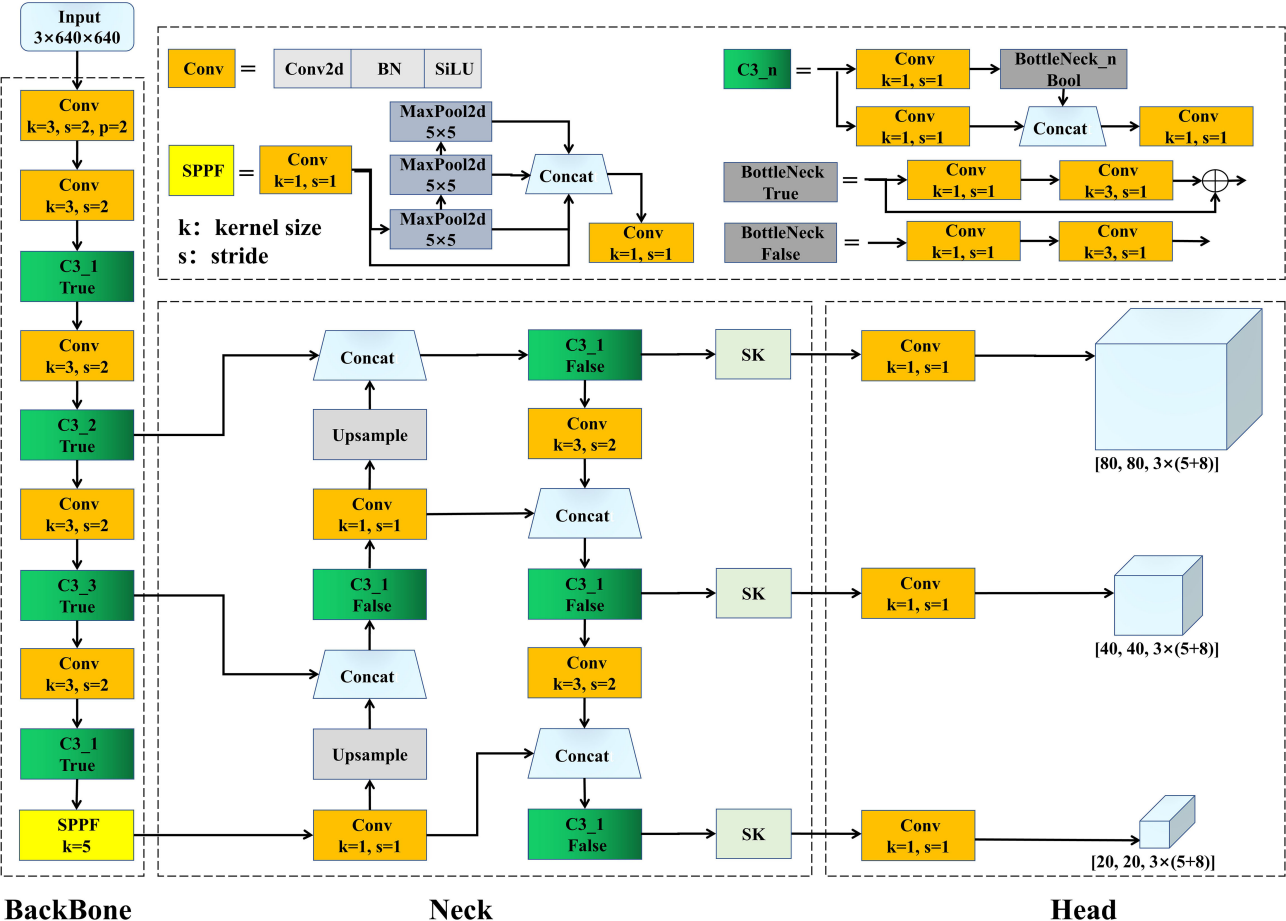
Improved YOLOv5s architecture.

In the context of the application requirements for embedded computing in apple harvesting equipment, the network model must possess the capability to rapidly and accurately identify apples (De-An et al., 2011). We chose YOLOv5s, which was designed specifically for embedded systems, for the baseline network model as it strikes a balance between detection speed and accuracy. YOLOv5s primarily consists of three components: the Backbone, Neck, and Head. To enhance the model’s performance, modifications were made to both the backbone and Neck sections; see the overall architecture depicted in **Figure 5**.

The backbone is responsible for transforming the input image into multi-layer feature maps suitable for object detection tasks. This component primarily consists of Conv modules, C3 modules, and Spatial Pyramid Pooling Fast (SPPF) modules. The Conv module encompasses convolution (Conv2d), Batch Normalization, and the SiLU activation function. The C3 module draws inspiration from DarkNet53 in YOLOv3 (Redmon and Farhadi, 2018), combined with the design philosophy of CSPNet (Wang et al., 2020), and includes three Conv and multiple Bottleneck modules. The Bottleneck module employs the residual structure from ResNet (He et al., 2015), primarily in two variations: the first path uses a 1 × 1 convolution to halve the channel number of the feature map before a 3 × 3 convolution extracts features, ensuring consistent input and output channel numbers; while the second path uses a direct shortcut for residual connection, thus achieving feature fusion. The other variation omits the feature fusion step when no shortcut is applied. The C3 module aims to enhance the network’s depth and receptive field, thereby improving its feature extraction capabilities. Inspired by SPPNet (He et al., 2014), the SPPF module replaces a large pooling kernel with multiple smaller ones, thereby enhancing the execution speed and feature expressiveness. In Sections 2.3.3 and 2.3.4, we describe a guided pre-training strategy based on self-supervised learning and knowledge transfer, developed with the aim of training a backbone capable of fine-grained feature extraction for multi-obstructed apple detection.

The Neck module is tasked with integrating feature maps at different levels, producing feature maps with multi-scale information, and forwarding those maps to the Head section. This component is composed of Conv modules, Upsample, Concat, and a C3 module without a shortcut. Moreover, the design of the Neck incorporates structures from FPN (Lin et al., 2016) and PAN (Liu et al., 2018), employing both top-down and bottom-up feature extraction methods, thus facilitating the fusion of shallow graphic features and deep semantic features of the network. In Section 2.3.5, we detail how the SK module is introduced to enhance the Neck’s focal representation capabilities for target region features. The Head primarily conducts multi-scale object detection on the feature maps integrated by the Neck. This module’s design aims to expand the channel numbers of the three differently sized feature maps in the Neck. The expanded channel number calculation method is presented in Equation 1.


(Eq. 1) CM=(OAC+5)×NA

where *OAC* represents the number of occluded apple categories, and the 5 represents five parameters: the bounding box center’s x- and y-coordinates, width, height, and confidence score). *NA* represents the number of anchors per detection layer. In this study, *OAC* is 8,and *NA* is 3.

#### 2.3.3 Pre-training of the student backbone model based on BYOL

In recent years, Self-Supervised Learning (SSL) has gained significant attention in the realm of image processing, offering a novel approach to model training that does not rely on manually annotated data (Jing and Tian, 2019). By autonomously extracting labels from a vast amount of unlabeled data, this training method substantially reduces the dependency on annotated data, leading to significant savings in both time and cost. Early SSL methods typically relied on designing specific predictive tasks, such as estimating image rotation angles or color arrangements, thereby encouraging the model to discern meaningful image features (Doersch et al., 2015). More recently, researchers have identified SSL techniques that draw similar image features closer while pushing dissimilar ones apart, such as Momentum Contrast (He et al., 2019), BYOL (Grill et al., 2020), and SimCLR (Chen et al., 2020b). Notably, BYOL stands apart from other contrastive learning methods that rely on negative samples; instead, BYOL learns image representations from two distinct image views derived from a target network and an online network, respectively. This strategy not only streamlines the learning process, but also achieves efficient feature representation without the use of any negative samples. Given the potential of SSL in deep learning, this study leverages BYOL to enhance the performance of the YOLOv5s backbone.

The initial step involved setting up the target network model and the online network model. The backbone of YOLOv5s was first selected as the online encoder. Subsequently, the weights of the online network model were cloned to produce the target encoder, the calculation method is presented in Equation 2.


(Eq.2) $$F\left(x\right)=W_{v5s_{B_{i}}}\left(x\right)$$

where *F*(*x*) is the feature tensor extracted from the input image and $W_{v5s_{B\_1}}$ , i∈{online,target} represents the type of encoder.

To enhance the encoder’s generalization capability, we devised a data augmentation strategy considering the characteristics of agricultural images. Initially, random cropping and horizontal flipping of the images were employed, supplemented with color adjustments and brightness/contrast modifications, succinctly termed Color Adjustment (CRAJ). The calculation method for generating augmented images from the original images is presented in Equation 3.


(Eq. 3) $$x_{k}=RandomCrop\left(RandomHorizontalFlip\left(CRAJ\left(x\right)\right)\right)$$

where *x_k_*, k∈{1,2} represents the augmented image.

Subsequently, construction of the projection head and predictor was carried out. Within the online network model, both the projection head and predictor are composed of a multi-layer perceptron (MLP). The prediction calculation method for the online network model is presented in Equation 4.


(Eq. 4) $$z_{online\_i}=W_{2}\sigma \left(W_{1}F\left(x_{i}\right)\right)$$

In the target network model, the projection head consists of a single MLP and does not include a predictor. The projection calculation method for the online network model is presented in Equation 5.


(Eq. 5) $$z_{target\_i}=\sigma \left(W_{1}^{'}F\left(x_{i}\right)\right)$$

where *W*_1_ and *W*_2_ represent the weights of the projection head and predictor in the online network model, respectively; $W_{1}^{'}$ denotes the weights of the projection head in the target network model; and σ is the ReLU activation function.

Subsequently, construction of the BYOL loss function was undertaken. The loss calculation method is presented in Equation 6.


(Eq. 6) $$L=\sum_{i=0}^{1}2-2\times \frac{\langle z_{online\_i},z_{target\_i}\rangle }{\text{||}z_{online\_i}\text{||}{\;}_{2}\times \text{||}z_{target\_i}\text{||}{\;}_{2}}$$

where the inner product of vectors is denoted by 〈·,·〉 , z_online_1_ represents the output processed by the online encoder when processing x_1_, z_target_1_ signifies the output processed by the target encoder when processing x_2_, and L is the result of the loss computation.

Subsequently, an overarching training optimization strategy for the network model was devised. Utilizing standard backpropagation and the Adam optimizer, the gradient of the loss function L with respect to the weights of the online encoder was computed, allowing for updating of the online weights. Concurrently, to stabilize the self-supervised training process, we employed an exponential moving average strategy to update the weights of the target encoder, which was calculated in Equation 7.


(Eq. 7) $$W_{target}=\beta \times W_{target}+\left(1-\beta \right)\times \left[W_{v5s_{B_{online}}},W_{1}\right]$$

where *W_online_* represents the combination of [ $W_{v5s_{B\_online}}$ , $W_{1}$ , *W*_2_] and *W_target_* represents [ $W_{v5s_{B\_target}}$ , $W_{1}^{'}$ ]. For β, a value of 0.90 was set to update the weights of the target encoder.

**Figure 6:**
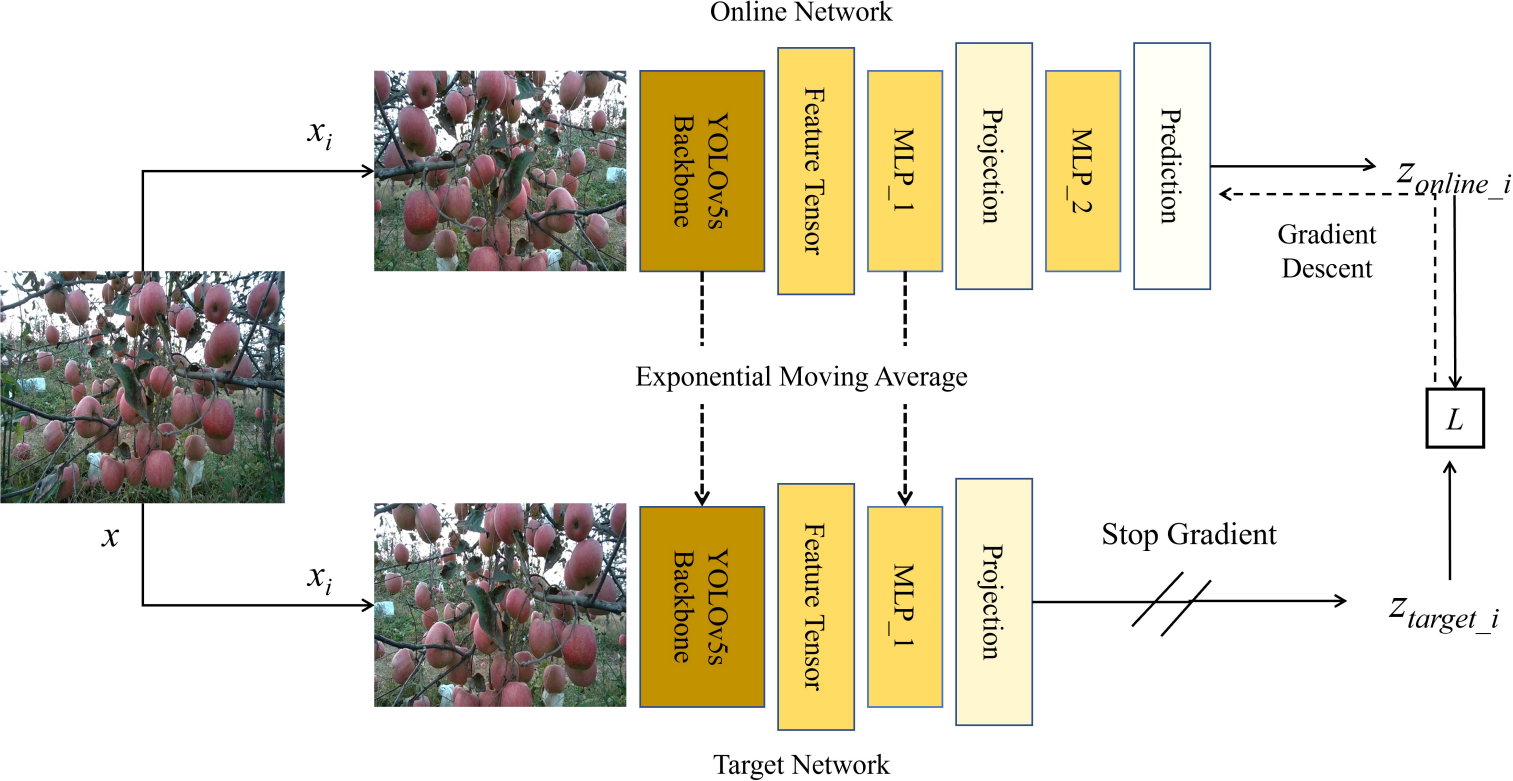
Self-supervised training framework of the YOLOv5s backbone based on BYOL.

The self-supervised training process of the YOLOv5s backbone based on BYOL is illustrated in **Figure 6**. We utilized 5000 images to deeply pre-train the backbone of YOLOv5s in a self-supervised manner. The BYOL method efficiently learns features while relying solely on the loss of the online network. Upon completion of the pre-training step, the acquired weights—encapsulating vital visual feature information about apple trees—were stored within the YOLOv5s backbone. These weights could then be applied to downstream object detection tasks. In the subsequent phase, we fine-tuned the YOLOv5s backbone using the test data set, resulting in the final BYOL-improved YOLOv5s.

#### 2.3.4 Pre-training of the student backbone model based on knowledge transfer

In the realm of deep learning, the process of knowledge transfer primarily refers to utilizing a model trained on one task as a starting point for training on another task (Passalis and Tefas, 2018). The foundational concept is to transfer the knowledge from the teacher backbone model to the student backbone model, with the hope that the student backbone model may approach or even surpass the performance of the teacher model (Chen et al., 2020a). We focused on enhancing the feature extraction capability of the YOLOv5s backbone, exploring how to further amplify the backbone’s feature extraction ability through knowledge transfer methods by leveraging the pre-trained YOLOv5s.

In orchard environments, apples are frequently occluded by leaves, branches, and other apples. It is crucial to consider how to enable the model’s backbone to learn about the shapes, sizes, and textures of such obstructions. We employed a backbone distillation approach, utilizing intermediate feature activation layers to enable the student backbone model to learn from the teacher model. These intermediate feature activation layers can accurately represent the shapes and textures of leaves, branches, and apples, thereby offering improved detection in scenarios with multiple obstructions. Given the inherently commendable performance of YOLOv5s and based on preliminary experimental results, we decided to use the backbone of YOLOv5s trained with supervised data as the teacher backbone model. We chose the untrained YOLOv5s backbone as the student backbone model. This design strategy aims to achieve self-guidance and transfer learning for YOLOv5s, thus promoting enhanced backbone performance. Throughout this process, multiple intermediate feature activation layers in the teacher backbone model are utilized. For each intermediate layer, denoted by l, we compute the corresponding feature activation results $F_{T}^{l}$ . To enable the student backbone model, denoted by *S*, to learn the information from these intermediate feature activation layers, we designed a feature matching loss, which was calculated in Equation 8.


(Eq. 8) $$L_{feature}^{l}=\frac{1}{N_{l}}\Sigma_{i=1}^{N_{l}}{\left|\left|F_{T}^{l,i}-F_{S}^{l,i}\right|\right|}_{2}^{2}$$

where N_1_ represents the number of feature channels in layer l, while $F_{T}^{l,i}$ and $F_{S}^{l,i}$ denote the feature tensors of the student and teacher backbone models at layer l in channel i, respectively. In our practical experiments, we selected the fifth feature activation layer as preliminary experiments indicated that the model’s backbone performance reached its peak when l is 5.

For the knowledge transfer process, we employed several techniques to ensure training stability and expedite convergence, including learning rate decay, early stopping strategies, and data augmentation. We configured the optimizer as Adam with an initial learning rate of 0.001 and weight decay of 0.0005. The learning rate was scheduled to decrease by 2% every 10 epochs.

**Figure 7:**
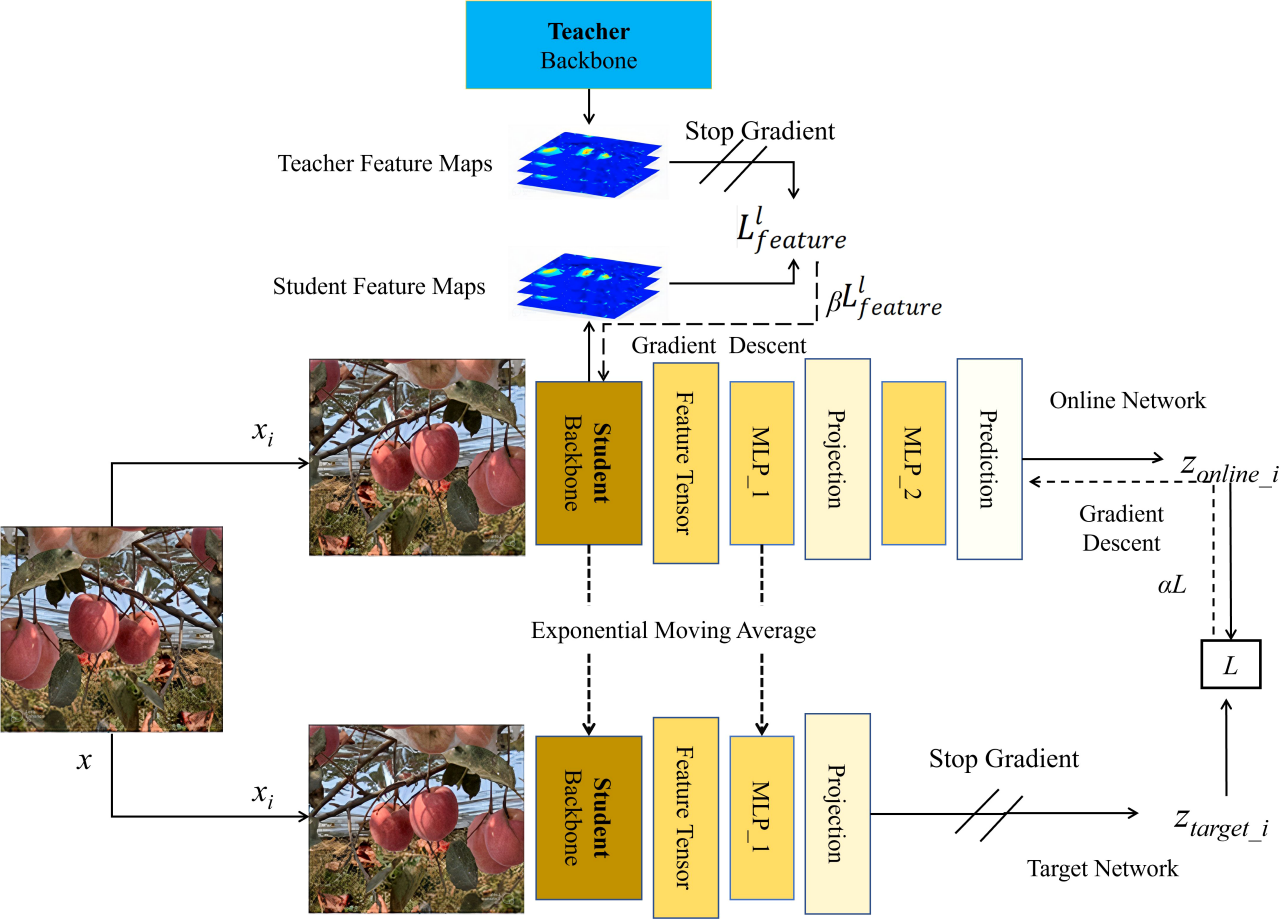
Guided training of the student backbone model through fusion of the teacher backbone model and BYOL.


To fully leverage the limited training data set and quantity of unlabeled data, we further explored combinations of self-supervised learning methods in addition to knowledge transfer, with the aim of enhancing the performance of the YOLOv5s backbone for improved results in object detection tasks. The specific architecture is illustrated in **Figure 7**, and the overall loss calculation method derived from the combination of these two approaches is presented in Equation 9.

(Eq. 9) $$L_{all}=\alpha L+\beta L_{feature}^{l}$$

where L represents the contrastive loss generated through self-supervised learning and ${\text{L}}_{\text{feature}}^{\text{l}}$ denotes the loss arising from knowledge transfer. Additionally, α and β are hyperparameters, with βcontrolling the strength of knowledge transfer and α regulating the impact of self-supervision on model training. In our experiments, these hyperparameters were set as 0.1 and 0.9, respectively.

#### 2.3.5 Selective Kernel module

Attention mechanisms have recently become indispensable in the design of deep learning models, especially when addressing intricate image problems (Zhang et al., 2018). The Squeeze-and-Excitation (SE) attention mechanism optimizes feature weights at the channel level (Hu et al., 2017), yet its responsiveness to specific spatial contexts remains limited. In contrast, CBAM aims to integrate both spatial and channel attention (Woo et al., 2018), but its performance still requires improvement when handling multi-scale and intricate occlusion scenarios. Given the demand for detecting apples with various types of occlusion—especially considering the sensitivity to diverse occlusion patterns and target size variations—a strategy that can dynamically adjust the receptive field has become crucial. Considering this need, the SK module has a unique advantage (Li et al., 2019): it endows each spatial location with the ability to dynamically select convolutional kernels, offering profound contextual understanding of different occlusion types, thereby achieving more refined and adaptive feature extraction.

**Figure 8:**
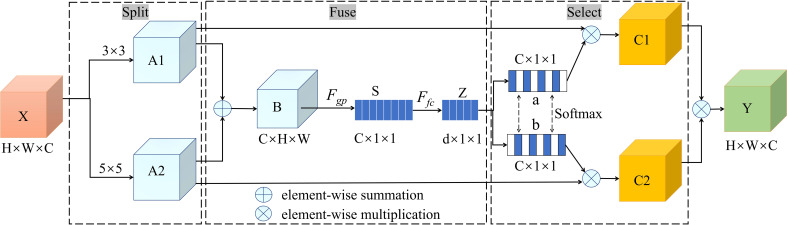
Selective kernel module.

The SK module is illustrated in **Figure 8**. In this model, the input feature tensor X first undergoes full convolution operations with two distinct kernel sizes. For this study, 3 × 3 and 5 × 5 convolutional kernels were employed, with dilation parameters set to 1 and 2, respectively, yielding two feature maps (denoted A1 and A2) matching the dimensions of the original feature map. Subsequently, the corresponding elements of A1 and A2 are summed to produce an overall feature map, B, which retains the dimensions of the original input feature map. B is then subjected to a global average pooling operation, resulting in the feature map S. A fully connected layer (FC) is then utilized to extract channel attention information, producing a further feature map Z, with dimensions of d × 1 × 1. Then, the feature map Z is separately processed by two softmax functions, a and b, to obtain the channel attention information. The channel attention information is then multiplied element-wise with the feature maps A1 and A2, outputting two channel attention feature maps, denoted C1 and C2. To further emphasize key features and suppress extraneous information, C1 and C2 are fused by adding their corresponding positions, yielding a final feature map Y, with dimensions H × W × C.

### 2.4 Model training and performance metrics

#### 2.4.1 Training equipment

We conducted all experiments on a deep learning server equipped with a 64-core Intel Xeon(R) Gold 6226R mailto:v4@2.90v4@2.90 Hz CPU, 251.6 GB of RAM, and a 1.9 TB solid-state drive, along with two 16 GB NVIDIA Tesla V100 GPUs. On the software side, the server ran the Ubuntu 20.04 operating system with NVIDIA driver version 495.46, PyTorch 1.10, CUDA 11.5, and cuDNN 8.2.4.

#### 2.4.2 Training details

We adopted the following training strategy. First, we performed teacher backbone model training based on YOLOv5s and a labeled data set of multi-occluded apples. This training ensured that the model could better understand and handle apple occlusion scenarios. Once the model converged, we saved the optimal weights and extracted the backbone weights for further use in training the student backbone model. Next, we extracted the backbone portion from the improved YOLOv5s model and integrated it into both the online and target backbones of BYOL. During this stage of training, while the teacher backbone was frozen, we iteratively updated the student backbone model using the self-supervised learning loss and knowledge transfer loss. After training on 5000 orchard images, we obtained an optimal student backbone model. Next, we swapped the optimal student backbone model with the improved YOLOv5s backbone and proceeded to fine-tune the model. Notably, the entire training process was divided into two stages: The backbone freezing stage and the backbone unfreezing stage. In the initial 100 iterations of the backbone freezing stage, the backbone parameters remained unchanged and we only fine-tuned the neck and head networks. The initial learning rate for this stage was set to 0.002, and we used the Adam optimizer with a momentum parameter of 0.85. If the loss did not decrease between two iterations, the learning rate was halved. After 100 iterations, we entered the backbone unfreezing stage, where all network parameters were updated. The initial learning rate was set to 0.001, and the learning rate update strategy was the same as in the previous stage. Ultimately, when the network model converged, we obtained the YOLOv5s backbone optimized for multi-occluded apples.

#### 2.4.3 Performance metrics

We evaluated the performance of the trained network model using four metrics: Precision (P), Recall (R), Average Precision (AP), and Mean Average Precision (mAP). The specific calculation methods for these metrics are presented in Equations 10–13.


(Eq. 10) P=TP/(TP+ FP)

(Eq. 11) R=TP/(TP+FN)

(Eq. 12) $$AP=\int_{0}^{1}P_{n}(R_{n})d\;R_{n}$$

(Eq. 13) $$mAP\left(n\right)=0.125\times \sum_{n=1}^{8}AP\left(n\right)$$

where P represents the proportion of correctly predicted boxes among all predicted boxes and R represents the proportion of correctly predicted boxes among all labeled boxes. To assess the model’s performance in different categories, we used AP(n), which denotes the average precision for the nth class of multi-occluded apples, and mAP, which represents the average precision across the eight types of occluded apples. Here, TP stands for the number of predicted boxes correctly matched with annotated boxes, FP represents the number of incorrectly predicted boxes, and FN represents the number of labeled boxes that are not predicted.

## 3 Results

### 3.1 Detection results and analysis

**Figure 9:**
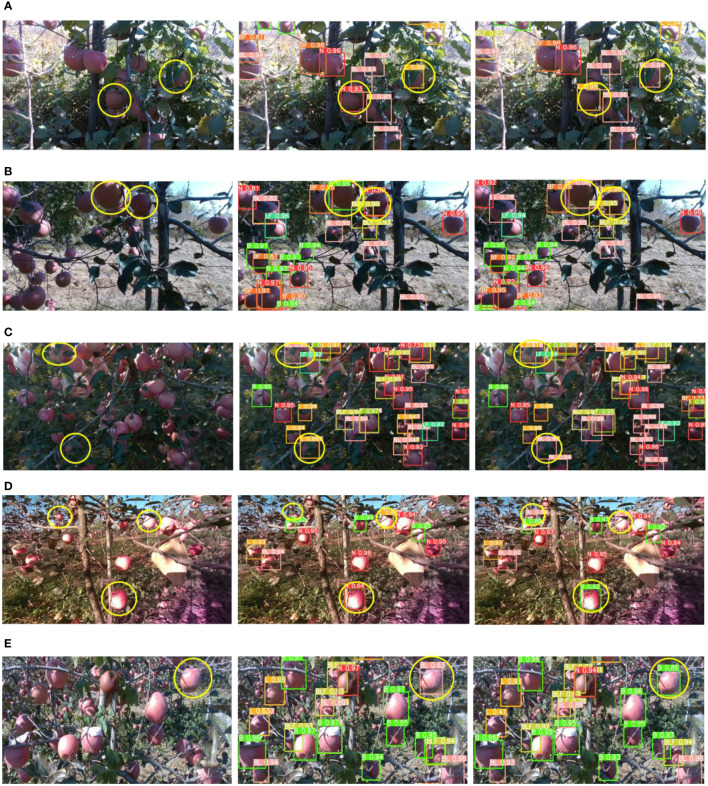
Recognition results before and after improvement of YOLOv5s. **(A)** Comparison of detection results under side-lit conditions. **(B)** Comparison of detection results under back-lit conditions. **(C)** Comparison of detection results under low-light conditions; **(D)** Comparison of detection results under front-lit condition 1. **(E)** Comparison of detection results under front-lit condition 2.

To precisely assess the performance of the improved YOLOv5s model in terms of apple graspability detection, validation was conducted on a test set comprising 560 images. For the evaluation process, three critical metrics were defined: AP_GA_, AP_TUGA_, and AP_UGA_, representing the average precision of detection for graspable, temporarily ungraspable, and ungraspable apples, respectively. **Table 2** presents a performance comparison between the improved YOLOv5s and the original YOLOv5s. Notably, when compared to the original network, the improved YOLOv5s exhibited increases of 2.08%, 3.03%, and 3.65% in the mAP, AP_GA_, and AP_UGA_ metrics, respectively, while showing a slight decline of 0.45% in the AP_TUGA_ metric. This result suggests that the improved YOLOv5s achieved enhanced detection accuracy for the GA and UGA categories, with only a minor decrease in performance for the TUGA category. **Figure 9** provides a comparative visualization of detection outcomes for both models, in which instances of misidentification by YOLOv5s are indicated by yellow circles. Ultimately, the improved YOLOv5s model achieved accurate discernment.

**Table 2:** Comparative detection performance results between YOLOv5s and improved YOLOv5s.

Network Model	mAP (%)	AP_GA_ (%)	AP_TUGA_ (%)	AP_UGA_ (%)	FLOPs(G)	FPS
YOLOv5s	91.29	91.55	92.20	90.13	16.4	120
Improved YOLOv5s	94.54	94.78	93.86	94.98	19.2	101

In image detection tasks conducted under various lighting conditions, the original YOLOv5s exhibited several misclassification errors. Specifically, under side lighting (**Figure 9A**), the L was misclassified as N and BL as L. These errors were primarily attributed to the subdued brightness of the apple leaves in shadowed areas towards the upper right, leading to indistinct leaf features and, consequently, misidentification of N. Additionally, substantial leaf occlusion diminished the salient characteristics of prominent branches, causing misidentification of BL. In the back-lit case (**Figure 9B**), low light occluded subtle features of L and BL, resulting in their misclassification as N and B, respectively. In low-lighting situations (**Figure 9C**), confusion between BL and L was observed. Under front-lit condition 1 (**Figure 9D**), similarities between background and target features resulted in detection failures. This inadequacy was a consequence of YOLOv5s losing certain features during the convolution and pooling processes. In addition, under front-lit condition 2 (**Figure 9E**), the shadow formed by the leaves on the apples led to B being mistaken for BL. The improved YOLOv5s model ameliorated the detection outcomes for all aforementioned tasks, yielding superior performance in terms of capturing fine-grained features. This result highlights the model’s enhanced ability to discern between similar categories. Overall, the improved YOLOv5s consistently excelled across diverse lighting conditions, fulfilling the perceptual needs of apple harvesting equipment more effectively and significantly mitigating the risk of misjudging apple graspability.

### 3.2 Ablation study

To validate the positive impact of each proposed improvement on the performance of the YOLOv5s model, we conducted ablation experiments, the results of which are presented in **Table 3**. During the training of YOLOv5s, we employed an online network self-supervised learning strategy based on BYOL. The purpose of this strategy was to enhance the feature extraction capabilities of the online network model with respect to the images. By introducing a teacher backbone model to train the student backbone model, we aimed to more accurately map the teacher feature space to the student feature space. Additionally, we integrated the SK module with the goal of optimizing the detection capabilities for occluded targets at different scales (including distance and size), thereby reducing instances of missed detections and errors.

**Table 3:** Results of the ablation experiments.

YOLOv5s	BYOL	KT	SK	mAP (%)	AP_GA_ (%)	AP_TUGA_ (%)	AP_UGA_ (%)	FLOPs(G)	FPS
√	×	×	×	91.29	91.55	92.20	90.13	16.4	120
√	√	×	×	93.04	93.30	93.05	92.77	16.4	120
√	√	√	×	93.77	93.82	92.60	93.90	16.4	120
√	×	×	√	93.71	94.08	93.32	93.73	19.2	101
√	√	√	√	94.54	94.78	93.86	94.98	19.2	101

*”×” indicates the module is not used, while “√” indicates the module has been used.

**Figure 10:**
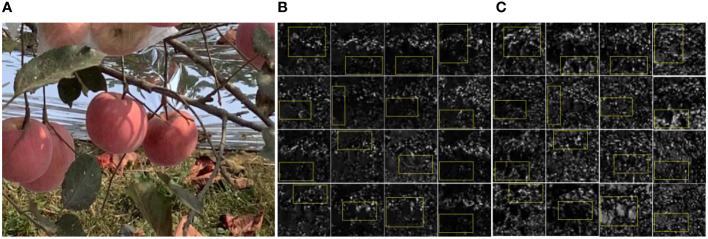
Backbone Feature maps of YOLOv5s Before and After Improvement. **(A)** Reference Image. **(B)** Backbone feature maps without BYOL. **(C)** Backbone feature maps with BYOL.

Following training on the YOLOv5s backbone under the BYOL self-supervised learning strategy, the backbone was integrated into the YOLOv5s model. Subsequently, YOLOv5s was fine-tuned using the test set. The end result was a YOLOv5s model reinforced through the BYOL self-supervised approach. The performance improvements in mAP, AP_GA_, AP_TUGA_, and AP_UGA_ were 1.75%, 1.75%, 0.85%, and 2.64%, respectively, indicating the enhanced ability of the backbone to extract the features of apples. The Reference image (**Figure 10A**) was selected to provide a visual comparative analysis of the backbone feature maps before and after BYOL training with YOLOv5s, as shown in **Figures 10B, C**. Additionally, specific attention was paid to the feature maps of the fifth layer. In post-training with the BYOL strategy, the convolutional layers indicated improved detection of the subtle contours and textures of branches and apples. The feature maps from this layer—in terms of both quality and extent—noticeably surpassed those from the original YOLOv5s model, providing solid evidence for the efficacy of the BYOL strategy in enhancing the fine-grained feature extraction capabilities of the YOLOv5s backbone.

We further evaluated the improvement of the backbone’s performance through a guided training strategy integrating BYOL approaches with knowledge transfer. Across the various evaluation metrics, performance increases of 0.73%, 0.52%, and 1.13% were observed in mAP, AP_GA_, and AP_UGA_, respectively. However, a decline of 0.45% was observed in AP_TUGA_. Both the computational complexity and inference speed of the network remained unaffected. We carefully examined the disparities between the multi-level feature maps of the hidden layers in the teacher backbone model and the student backbone model in order to compute the regularization loss. This loss was successfully integrated with the self-supervised learning loss to iteratively update the student backbone model. Notably, while the teacher backbone model was trained based on a self-supervised learning approach using YOLOv5s, the teacher backbone model still offers beneficial guidance on the hidden features of the student backbone model. This guidance is possibly due to the supervisory signals generated by the teacher backbone model, which provide a clear learning direction for the student backbone model at the same scale. This positively influenced the convergence process of the student backbone model.

**Figure 11:**
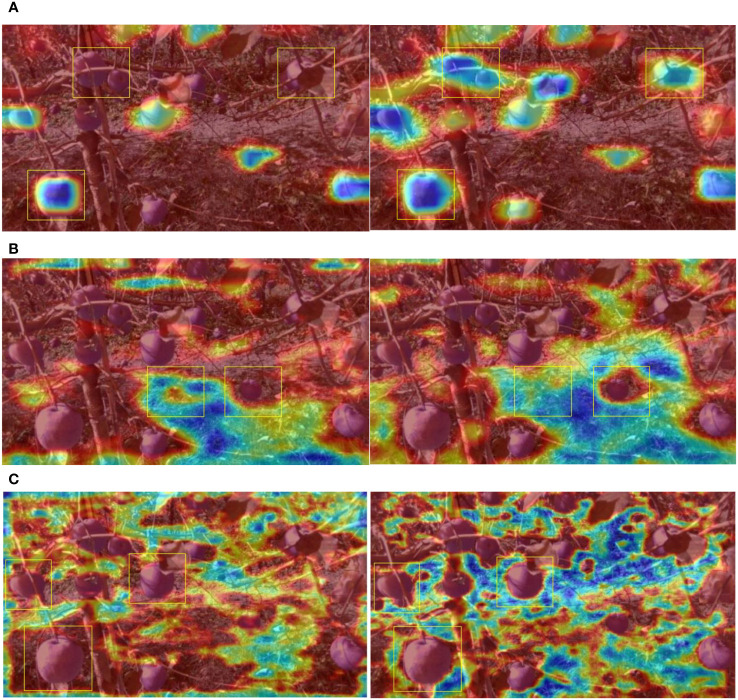
Visualization of features maps before and after addition of the SK module. **(A)** Comparison of the visualization results for 20 × 20 feature maps with and without the SK module. **(B)** Comparison of visualization results for 40 × 40 feature maps with and without the SK module. **(C)** Comparison of the visualization results for 80 × 80 feature maps with and without the SK module.

Upon integrating only the SK module into YOLOv5s, further performance enhancement was realized. Specifically, the improved model experienced increases of 2.42%, 2.53%, 1.12%, and 3.6% in mAP, AP_GA_, AP_TUGA_, and AP_UGA_, respectively. Although inclusion of the SK module led to a computational complexity increase of 2.8 GFLOPs, the computational speed still adequately met real-time processing requirements. To elucidate the reasons for this performance enhancement, the output feature maps of the detection network model across three sizes were mapped to pseudocolor images in the original size and overlaid onto the original images, allowing for visualization of the output features. These visualized feature images were generated in three resolutions: 80 × 80, 40 × 40, and 20 × 20, as depicted in **Figure 11**. In **Figure 11A**, the 20 × 20 resolution feature map primarily highlights the higher-order features of the apples while simultaneously smoothing out background details. This representation aids in more accurately distinguishing between background and target apples during detection. With the introduction of the SK module, one can directly observe a pronounced enhancement in the model’s apple perception capabilities, thus reducing omissions in apple detection. The particular feature map shown in **Figure 11B** primarily accentuates the background. Before the introduction of the SK module, the extracted landmarks were somewhat coarse; however, with the SK module, there was a significant expansion in the model’s feature perceptive range. In **Figure 11C**, the 80 × 80 feature map reveals more profound background perception and heightened differentiation between all apple features, thereby validating that integration of the SK module justifiably and effectively elevated the performance of the improved model.

## 4 Discussion

In the complex environments characteristic of orchards, harvesting equipment needs to not only precisely detect the locations of fruits but also intelligently determine the types of obstruction preventing access to the fruit. It is crucial to discern the fruit’s graspability to help such equipment avoid rigid obstructions and achieve damage-free harvesting of apples. As shown in **Table 4**, the methodology proposed in this study achieved scores of 94.54%, 94.78%, 93.86%, and 94.98% in the mAP, AP_GA_, AP_TUGA_, and AP_UGA_ metrics, respectively. These results demonstrate that the proposed approach provides robust support for both actual harvesting needs and future research in related domains. To specifically discuss the advantages and underlying reasons for the improved performance of YOLOv5s over contemporary similar models, we conducted comparative experiments with the improved YOLOv5s and other popular object detection network models. Additionally, YOLOv5x was incorporated to observe the peak performance of the YOLOv5 series, serving as a benchmark for optimal performance. Lightweight network models primarily include YOLOX (Ge et al., 2021), YOLOv4-s (Bochkovskiy et al., 2020), and YOLOv7s (Wang et al., 2022). To ensure fairness and consistency in testing, a uniform data set was employed to deeply train and assess the performance of multiple network models. **Table 4** provides the detection results, detailing not only the Floating Point Operations (FLOPs) of each model, but also the processing speed advantages and disadvantages of each model, represented in terms of Frames Per Second (FPS).

**Table 4:** Test results for different network models.

Network Model	mAP (%)	AP_GA_ (%)	AP_TUGA_ (%)	AP_UGA_ (%)	FLOPs(G)	FPS
YOLOv5x	95.00	95.36	94.60	95.04	205.5	59
YOLOv5s	91.29	91.55	92.20	90.13	16.4	120
YOLOv4-s	88.40	87.64	89.65	87.91	15.4	164
YOLOv7s	72.20	70.56	71.94	74.10	13.2	113
YOLOX-s	90.40	91.56	89.85	89.79	26.8	73
improved YOLOv5s	94.54	94.78	93.86	94.98	19.2	101

The results indicated that, due to its larger weights, the YOLOv5x model distinguished itself from the many evaluated models, especially in the four evaluation metrics mAP, AP_GA_, AP_TUGA_, and AP_UGA_. However, the differences between the improved YOLOv5s and YOLOv5x on these key indicators were relatively minimal (0.46%, 0.58%, 0.74%, and 0.06%, respectively). These findings provide a critical insight: in the domain of graspable apple detection, the improvements introduced in this paper enabled YOLOv5s to achieve performance nearly on par with that of the YOLOv5x model. In terms of detection speed, the improved YOLOv5s significantly surpassed YOLOv5x, offering a distinct advantage for deployment in actual embedded devices. When juxtaposed with other prevalent lightweight network models, the improved YOLOv5s exhibited conspicuous performance enhancements in the mAP metric over YOLOv4-s, YOLOX-s, YOLOv7s, and YOLOv5s (by 3.25%, 4.5%, 22.32%, and 6.14%, respectively). Although YOLOv4-s presented outstanding inference speeds—reaching up to 164 FPS—its performance in various AP metrics was less than ideal, with results akin to those of YOLOv7s. This result offers a salient lesson: it is imprudent to solely prioritize speed at the expense of accuracy. Conversely, while YOLOv7s possesses a straightforward model structure, its overall performance was relatively underwhelming, suggesting that this model may not be appropriate for high-precision granular detection tasks. The integration of the SK module, despite enhancing the model’s computational demands, impacted its inference speed. However, the authors in (Suo et al., 2021) determined that the picking time for a singular apple is approximately 2780 milliseconds. This result suggests that, even with a slight decrease in detection speed, our model remains adept at meeting the real-time requirements of agricultural apple harvesting equipment.

In summary, the advantages of the method proposed in this study were apparent in three primary areas. Initially, the proposed training approach and enhancement strategies for the network model enabled precise identification of various apple occlusion types within images. This method not only allows for determination of the graspability of apple targets, thus saving data annotation costs, but also achieved the stipulated design objectives. Furthermore, the detection performance of the improved YOLOv5s was markedly superior when compared to similar algorithms, making it well-suited to the damage-free harvesting needs of apple-picking equipment. The improved YOLOv5s retained its lightweight attributes, suggesting its significant potential for deployment in embedded hardware systems and laying a foundation for broader applications. On the other hand, certain limitations to our approach were identified. For example, the training process for our network model is intricate. Compared to the training protocol of the original YOLOv5s, this backbone requires multiple training iterations, prolonging the training duration. Additionally, the methodological data sets employed in this research largely prioritized red apples, leading to potential compromises in detection efficacy for non-red varieties, such as yellow and green apples. Finally, our detection strategy does not account for the potential impacts of fruit pose variations on apple graspability.

## 5 Conclusions

In response to the demand for more efficient and safe apple harvesting equipment, we proposed an improved YOLOv5s-based multi-occluded apple detection network model, which can efficiently identify graspable, temporarily ungraspable, and ungraspable apples. By incorporating knowledge transfer and BYOL strategies, along with integration of the SK module, the improved YOLOv5s model achieved optimized detection performance. Experimental data confirmed that this model offers strong performance in detecting multi-occluded apples, obtaining AP_GA_, AP_TUGA_, and AP_UGA_ scores of 94.78%, 93.86%, and 94.98%, respectively; furthermore, compared to the original YOLOv5s, our model presented improvements of 3.23%, 1.66%, and 4.85%, respectively, for these metrics. Although our proposed SK module slightly increased the computational complexity, it significantly enhanced detection accuracy and discrimination while still meeting the speed requirements for practical harvesting. When compared to state-of-the-art popular lightweight network models, the improved YOLOv5s model presented clear advantages in detection accuracy and approached the performance level of larger network models such as YOLOv5x. For future research, we intend to focus on integrating fruit occlusion types with fruit poses in the detection model, in order to further enhance the model’s practical value.

## Data availability statement

The raw data supporting the conclusions of this article will be made available by the authors, without undue reservation.

## Author contributions

HBL: Writing – original draft, Writing – review & editing, Conceptualization, Formal Analysis, Methodology, Supervision, Validation. PY: Formal Analysis, Methodology, Writing – review & editing. HYL: Formal Analysis, Writing – review & editing. XL: Investigation, Writing – review & editing. JQ: Project administration, Resources, Writing – original draft. QY: Formal Analysis, Investigation, Supervision, Conceptualization, Writing – review & editing. CG: Resources, Visualization, Writing – original draft. YS: Conceptualization, Funding acquisition, Writing – review & editing.
